# The Antineoplastic Effect of Dimethyl Fumarate on Virus-Negative Merkel Cell Carcinoma Cell Lines: Preliminary Results

**DOI:** 10.3390/cancers15020547

**Published:** 2023-01-16

**Authors:** Thilo Gambichler, Lyn G. Brüggestrat, Marina Skrygan, Christina H. Scheel, Laura Susok, Jürgen C. Becker

**Affiliations:** 1 Skin Cancer Center, Department of Dermatology, Venereology and Allergology, Ruhr-University Bochum, 44791 Bochum, Germany; 2 Translational Skin Cancer Research, German Cancer Consortium (DKTK) Partner Site Essen, Düsseldorf, Department of Dermatology, University Duisburg-Essen, 45117 Essen, Germany; 3 Deutsches Krebsforschungszentrum (DKFZ), 69120 Heidelberg, Germany

**Keywords:** merkel cell carcinoma, fumaric acid esters, dimethyl fumarate, monomethyl fumarate

## Abstract

**Simple Summary:**

Merkel cell carcinoma (MCC) is an aggressive skin cancer with limited treatment options for the advanced disease once immunotherapy has failed. MCC carcinogenesis has been attributed to the integration of an oncogenic virus or chronic UV radiation. The antineoplastic effects of dimethyl fumarate (DMF) have recently been shown in melanoma. Three virus-negative UV-associated MCC cell lines were treated with different doses of DMF. The cytotoxic effects and cell proliferation were assessed by the MTT cytotoxicity assay and BrdU proliferation assay for four different treatment periods. The reductions in cell viability and proliferation were demonstrated for all the cell lines used, with DMF proving to be significantly effective.

**Abstract:**

Merkel cell carcinoma (MCC) is a rare, difficult-to-treat skin cancer once immunotherapy has failed. MCC is associated either with the clonal integration of the Merkel cell polyomavirus (MCPyV) or mutagenic UV-radiation. Fumaric acid esters, including dimethyl fumarate (DMF), have been shown to inhibit cell growth in cutaneous melanoma and lymphoma. We aimed to explore the effects of DMF on MCPyV-negative MCC cell lines. Three MCC cell lines (MCC13, MCC14.2, and MCC26) were treated with different doses of DMF. The cytotoxic effects and cell proliferation were assessed by the MTT cytotoxicity assay and BrdU proliferation assay at different time points. A significant reduction in cell viability and proliferation were demonstrated for all the cell lines used, with DMF proving to be effective.

## 1. Introduction

Merkel cell carcinoma (MCC) is an uncommon cutaneous malignancy with epithelial and neuroendocrine differentiation. MCC cells share morphological, immunohistological, and ultrastructural features with Merkel cells. However, there is no obvious direct histogenetic link between MCC and Merkel cells. Importantly, Merkel cell polyomavirus (MCPyV) is clonally integrated into about 80% of the MCC in patients of the Northern hemisphere. By contrast, MCPyV-negative MCC is characterized by a high mutational burden similar to other ultraviolet (UV)-associated skin tumors such as cutaneous melanoma. Nevertheless, the distinct molecular pathogenesis of MCC and its link to MCPyV is not yet fully understood. The biological behavior of MCC is highly aggressive with high rates of local recurrences, regional lymph node, and distant metastasis. When compared to the chemotherapy era, the management of advanced MCC has significantly been improved since the introduction of immune checkpoint inhibitors. Nevertheless, only about 50% of patients with MCC benefit from immunotherapy [1,2,3,4,5,6,7].

Fumaric acid esters (FAE) are successfully used for the treatment of psoriasis and multiple sclerosis. Their antioxidative, immunomodulatory, and neuroprotective properties cause FAE to be attractive therapeutic candidates for other conditions as well. Currently, four FAE formulations are approved as human drugs: monomethyl fumarate (Bafiertam^™^), dimethyl fumarate (DMF, Tecfidera^®^, Skilarence^®^, and Fumaderm^®^), diroximel fumarate (Vumerity^™^), and zinc, magnesium, and calcium salts of monoethyl fumarate (Fumaderm^®^). DMF was first shown to possess cytoprotective and antioxidant effects in non-cancer models, which appeared to be related to the induction of the nuclear factor erythroid 2 (NF-E2)-related factor 2 (NRF2) pathway which also crosstalks with the NF-κB pathway [8,9,10,11,12,13].

Recently, it has been shown that the anticancer mechanisms of DMF are dose-dependent and are paradoxically related to the decrease in the nuclear translocation of NRF2. Several investigations indicate also the potential role of DMF in cancers, which are dependent on the NRF2 antioxidant and cellular detoxification program, such as KRAS-mutated lung adenocarcinoma. The antitumor effects of DMF have been shown in a variety of cancers, for example, in lymphomas, lung and breast cancer, and glioblastoma [14,15,16,17,18]. The transcription factor NRF2 orchestrates a cytoprotective inducible program, which counteracts the damaging effects of solar UV, an important etiological factor in skin cancers including cutaneous squamous cell carcinoma, MCC, and melanoma. Regarding the latter it has been shown that DMF inhibits the nuclear entry of NF-κB/p65, thus inhibiting melanoma cell invasion and metastasis [19,20,21,22,23,24,25,26,27]. Recently, Zhao et al. [28] identified MCPyV small T antigens-induced non-canonical NF-κB signaling as an essential tumorigenic pathway in MCC indicating that the inhibition of NF-κB signaling might be a promising treatment approach for MCPyV-positive MCC patients. Indeed, these results suggest that DMF is potentially useful as an anti-metastatic agent for the treatment of melanoma and possibly other skin cancers as well. Hence, we aimed to assess, for the first time, the antineoplastic activity of DMF in cultured MCPyV-negative MCC cell lines.

## 2. Materials and Methods

### 2.1. Cell Lines and Cultivation Methods

Three human MCPyV-negative MCC cell lines were used: MCC13 (source: Cell Bank Australia reference number: CBA-1338), MCC14.2 (source: CBA-1340), and MCC26 (source: CBA1341). On the basis of a material transfer agreement, all the cell lines have previously been provided to J.C. Becker by J.H. Leonard (Queensland Radium Institute Laboratory, Queensland Institute of Medical Research, Brisbane, Australia). The cells were cultured in RPMI 1640 with 10% fetal calf serum, 2 mM L-glutamine, and a penicillin (100 U/mL)/streptomycin (100 U/mL) mixture. All the substances were purchased from PAN-Biotech, Germany. The cells were grown at 37 °C, 95% humidity, and 5% CO_2_ as a monolayer to 60–80% confluency.

### 2.2. Treatment

The DMF was provided by Almirall Hermal GmbH (Reinbek, Germany). The DMF with high purity was dissolved in DMSO and sterile filtered. All three cell lines were treated with the prepared substance in different concentrations for different treatment periods (2 h, 6 h, 24 h, and 48 h). After the treatment time, the assay-specific procedures and measurements were performed. 

### 2.3. MTT Cytotoxicity Assay

To analyze the antineoplastic effects of the substance DMF on cell viability and to determine its dose–response relationship, colorimetric MTT cytotoxicity assays were performed on all three cell lines. The cells were treated with increasing concentrations of DMF in RPMI 1640 medium for 2 h, 6 h, 24 h, or 48 h. After the end of the incubation period, the yellow-colored MTT reagent 3-(4,5-dimethylthiazol-2-yl)-2,5-diphenyltetrazolium bromide (5 mg/mL) was administered to the cells. The MTT is reduced via mitochondrial dehydrogenase by metabolically viable cells to violet-colored formazan crystals that can be measured using photometry. The cell viability was analyzed using a microplate absorbance reader (ASYS, UVM340, Anthos Mikrosysteme GmbH, Germany/TECAN Infinite M-Plex reader) measuring the optical density. The amount of purple formazan crystals is directly proportional to the number of viable cells. All the MTT assays were performed with eight replicates. 

### 2.4. BrdU Proliferation Assay

In order to analyze and quantify the antiproliferative effect of the substance DMF, a BrdU (5-bromo-2-deoxyuridine) proliferation assay (Roche Applied Science, Mannheim, Germany) was carried out with all cell lines according to the manufacturer’s instructions. This proliferation assay is a non-isotopic colorimetric immunoassay to quantify BrdU incorporation into newly synthesized DNA from actively proliferating cells. All the experiments were set up according to the same scheme as in the MMT assay. The extent of the cell proliferation was determined via optical density using a microplate absorbance reader (ASYS, UVM340, Anthos Mikrosystheme GmbH, Germany/TECAN Infinite M-Plex reader). All the BrdU assays were performed with eight replicates.

### 2.5. Statistics

The statistical analyses were performed using the software package GraphPad Prism Version 9.4.1. Based on the assumption of a normal distribution for naturally measured values, an ordinary one-way ANOVA was used to calculate the significance. For a comparison between the individual measured values, a Holm-Šídák test was connected. If possible, the measured values were used to estimate an IC50.

## 3. Results

### 3.1. MTT

Based on previously published papers [14,15,16,17,18,19,20,21,22], which addressed DMF as a treatment reagent for cancer cell lines, we followed the dosages used and performed preliminary experiments to find a suitable concentration range. A range from 10 µmol/L to 200 µmol/L was selected. The three cell lines were seeded at different previously identified concentrations. MCC13 was seeded at 30.000 cells/well, MCC14.2 was seeded at 20.000 cells/well, and MCC26 was seeded at 50.000 cells/well. To provide the results not only as absorbance values, but to achieve a better comparability, the cell viability was expressed as a survival rate (SR) in percent; for this purpose, the negative control was set to 100% and the remaining values were put in relation to it.

**Figure 1:**
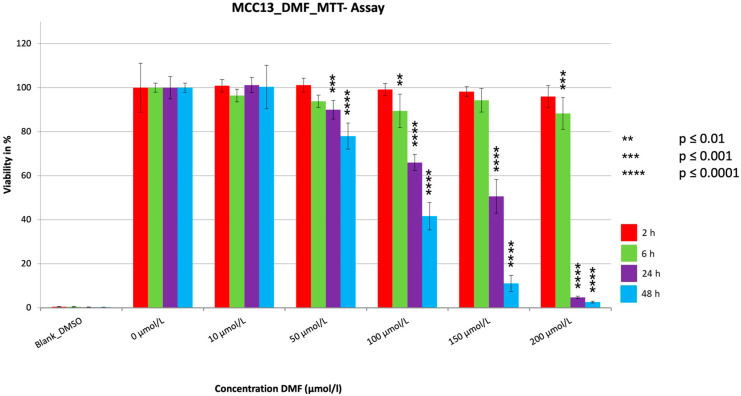
MTT measurement of the cell line MCC13 with a cell concentration of 30.000 cells/well with the addition of the substance DMF in different concentrations (10–200 µmol/L). Values are expressed as the mean ± SD of eight technical replicates. A multiple comparison test following the ANOVA was used to test whether there were significant differences between the individual mean values and the mean value of the normal control.

**Figure 2:**
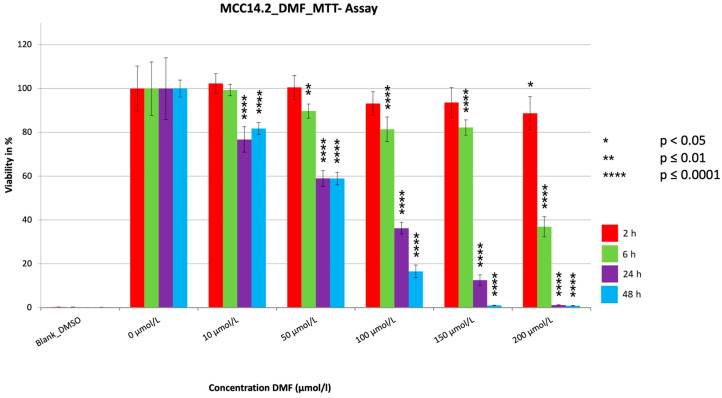
MTT measurement of the cell line MCC14.2 with a cell concentration of 20.000 cells/well with the addition of the substance DMF in different concentrations (10–200 µmol/L). Values are expressed as mean ± SD of eight technical replicates. A multiple comparison test following the ANOVA was used to test whether there were significant differences between the individual mean values and the mean value of the normal control.

**Figure 3:**
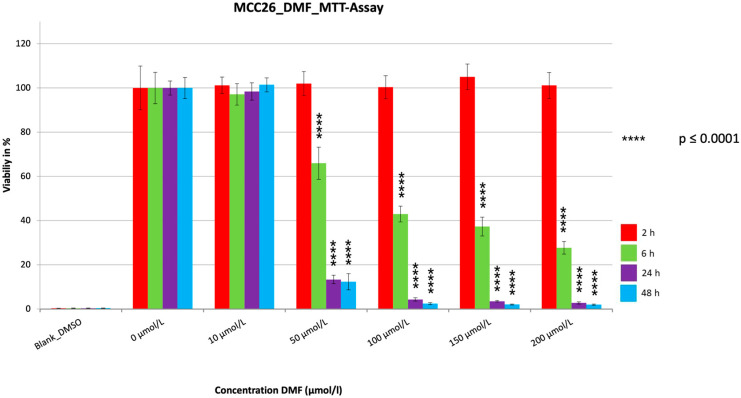
MTT measurement of the cell line MCC26 with a cell concentration of 50.000 cells/well with the addition of the substance DMF in different concentrations (10–200 µmol/L). Values are expressed as the mean ± SD of eight technical replicates. A multiple comparison test following the ANOVA was used to test whether there were significant differences between the individual mean values and the mean value of the normal control.

For all three cell lines, a very significant reduction in cell viability (*p* < 0.0001) was achieved (Figure 1, Figure 2 and Figure 3). Strikingly, after two hours of treatment, one could see in all three cell lines no (MCC13 and MCC26) or only a small (MCC14.2) but significant reduction (*p* = 0.0111) at the highest concentration used. The reduction was dependent on the dosage used as well as the duration of the treatment time. The strongest reductions were visible for the 24 h and 48 h treatment of each cell line. In this regard, the SR was reduced to 2.6% (MCC13), 0.8% (MCC14.2), and 2.0% (MCC26) after 48 h of treatment with 200 µmol/L. However, similarly low levels below 5% SR were also seen in all three cell lines after only 24 h. Notably, the SR was already reduced below 5% in cell line MCC26 at a concentration of 100 µmol/L compared to 150 µmol/L (MCC14.2) and 200 µmol/L (MCC13). Additionally, when comparing the IC50_48 h_ values of the three different cell lines it could be observed that MCC26 (31.60 µmol/L) was more sensitive than MCC14.2 (67.99 µmol/L) and MCC13 (97.80 µmol/L). 

### 3.2. BrdU

The concentrations of DMF used were derived from the previously performed MTTs (0 µmol/L–200 µmol/L). The three cell lines were seeded at different previously identified concentrations. MCC13 was seeded at 30.000 cells/well. MCC14.2 was seeded at 20.000 cells/well and MCC26 at 50.000 cells/well. To cause the results to be more descriptive and comparable, a proliferation rate (PR) in percent was calculated. For this purpose, the negative control was set as 100% and the other values were related to it. 

**Figure 4:**
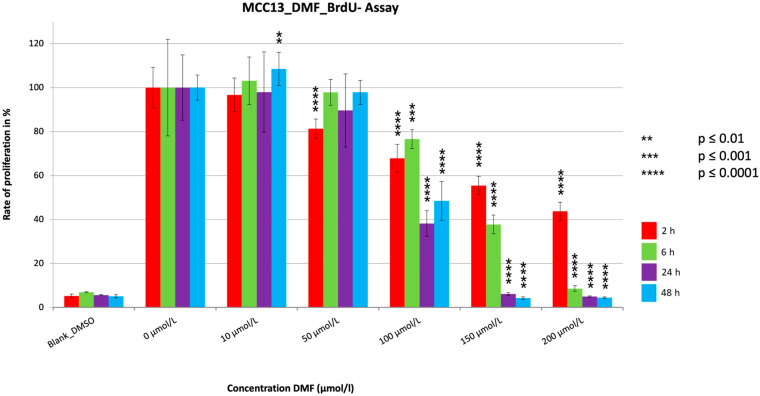
Cell proliferation measurements of the cell line MCC13 with a cell concentration of 30.000 cells/well with the addition of the substance DMF in different concentrations (10–200 µmol/L). Values are expressed as the mean ± SD of eight technical replicates. A multiple comparison test following the ANOVA was used to test whether there were significant differences between the individual mean values and the mean value of the normal control.

**Figure 5:**
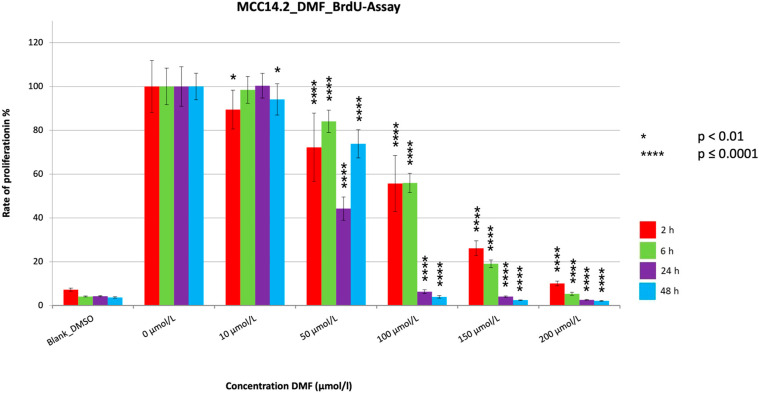
Cell proliferation measurements of the cell line MCC14.2 with a cell concentration of 20.000 cells/well with the addition of the substance DMF in different concentrations (10–200 µmol/L). Values are expressed as the mean ± SD of eight technical replicates. A multiple comparison test following the ANOVA was used to test whether there were significant differences between the individual mean values and the mean value of the normal control.

**Figure 6:**
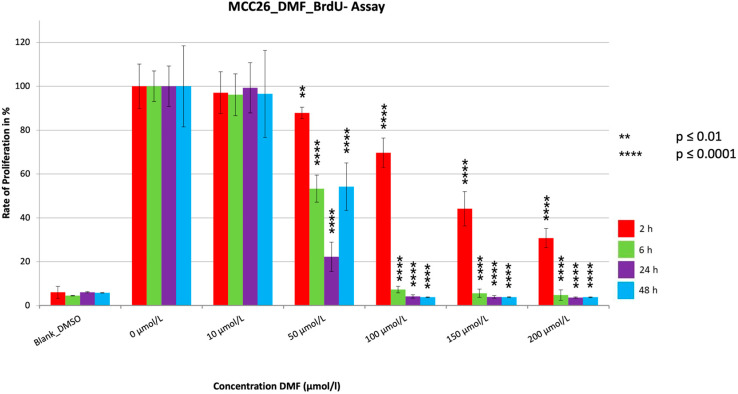
Cell proliferation measurements of the cell line MCC26 with a cell concentration of 50.000 cells/well with the addition of the substance DMF in different concentrations (10–200 µmol/L). Values are expressed as the mean ± SD of eight technical replicates. A multiple comparison test following the ANOVA was used to test whether there were significant differences between the individual mean values and the mean value of the normal control.

The proliferation assays showed a very significant reduction (*p* ≤ 0.0001) in PR for all the treatment durations (Figure 4, Figure 5 and Figure 6). The PR decrease was dose dependent. Despite a few variations, a tendency for PR to decrease with increasing treatment time could be seen for all cell lines, similar to the MTT assays. The extent of the reduction in PR among the three cell lines differed most during the two hour treatment period, with reductions seen to 10.1% (MCC14.2), to 30.8% (MCC26), and to 43.7% (MCC13). In contrast, the maximum reductions achieved were similar at 2.2% (MCC14.2), 3.7% (MCC26) and 4.2% (MCC13). A strong and significant reduction (PR < 5%) was achieved from a concentration of 100 µmol/L (MCC14.2, MCC26) and from 150 µmol/L (MCC13) for the 48 h treatment duration. Comparing the IC50_48 h_ values of the cell lines, it could be seen that, similar to the MTTs, the cell line MCC13 was the least sensitive with IC50_48 h_ being 99.53 µmol/L compared to 59.11 µmol/L (MCC14.2) and 50.53 µmol/L (MCC26).

## 4. Discussion

The data on the use of FAE in the treatment of cutaneous malignancies are limited. Most data are available for melanoma, which is an aggressive cancer characterized by a high mutational burden frequently affecting UV-associated genes, similar to the small subset of MCPyV-negative MCC [19,20,21,22,23,24,25,26,27]. For example, Takeda et al. [19] recently demonstrated that DMF decreases the cell viability, migration, and invasion of melanoma cells in vitro. Moreover, they could show that DMF inhibits spontaneous metastasis and tumor growth in an in vivo model. Their functional studies indicated that DMF prevents the nuclear translocation of NF-κB [19]. In addition, DMF inhibits the expression of matrix metalloproteinases and very late antigens. Furthermore, Takeda et al. [19] demonstrated that DMF decreases the expression of survivin and Bcl-extra large proteins. Previous studies also indicate anti-melanoma effects due to DMF-induced NF-κB inhibition [23,27]. Moreover, Valero et al. [21] showed in a SCID mouse model that combined treatment with dacarbazine and DMF efficiently reduces melanoma lymph node metastasis.

NF-κB pathway includes a transcription factor family that is responisble for the regulation of significant biological processes, including immune responses, inflammation, survival, cell cycle, and apoptosis. Two NF-κB pathways exsist in cells, say, the canonical and non-canonical pathway, which are differently activated. A variety of external stimuli, which are involved in cell proliferation, survival, inflammation, and immune responses, can induce the canonical NF-κB pathway. The activation of the IĸB kinase complex is a crucial step in the induction of the canonical NF-κB. By contrast, the tumor necrosis factor superfamily receptors activate the non-canonical NF-κB pathway. In the non-canonical NF-κB pathway, he NF-κB inducing kinase represents the main factor that is regulated by tumor necrosis factor receptor-associated factor 3-dependent degradation. The increased NF-κB activation observed during carcinogenesis is a result of an inflammatory microenvironment or direct mutations in NF-κB genes/oncogenes that regulate NF-κB signaling [29,30]. The involvement of non-canonical NF-κB signaling in virus-induced tumorigenesis was first suggested by the finding that this pathway is targeted by the human T-cell lymphotropic virus type 1-encoded oncoprotein TAX stimulating the activation of both canonical and non-canonical NF-κB pathways. The activation of non-canonical NF-κB by TAX is particularly unique for human T cell lymphotropic virus type 1-infected T lymphocytes, since normal T cells usually elicit only the canonical NF-κB pathway upon stimulation by the T cell receptor signal. Apart from human T cell lymphotropic virus type 1, several other human oncogenic viruses can also activate NF-κB, including the Kaposi sarcoma-associated human herpes virus (HHV) and the Epstein Bar virus. HHV-8 is tightly associated with Kaposi sarcoma, a malignancy frequently seen in individuals infected with HIV. In addition, HHV-8 is also found in several lymphoproliferative disorders, such as primary effusion lymphoma. EBV is well-known to persistently infect most healthy adults without normally causing overt diseases. However, accumulating evidence suggests that EBV may be associated with certain forms of lymphomas under immunodeficient conditions. EBV-encoded membrane protein LMP1 is a major viral activator of NF-κB that targets both the canonical and non-canonical pathways [29]. 

In line with the aforementioned data, Maeta et al. [31] recently observed that DMF inhibits proliferation and induced apoptosis in human T cell leukemia virus type 1-infected and transformed T cell lines (MT1 and MT2) by activating the poly-ADP-ribose polymerase. Furthermore, DMF inhibited the constitutive activation of both canonical and non-canonical NF-κB pathways in MT2 cells and the non-canonical NF-κB pathway in MT1 cells. DMF also downregulated anti-apoptotic proteins such as c-IAP2 and survivin in both cell lines. The data of Maeta et al. [31] indicate that DMF inhibits the proliferation and induces apoptosis in human T cell leukemia virus type 1-infected and transformed T cells by suppressing NF-κB signaling pathways. Nicolay et al. [18] also showed that DMF restores apoptosis in cutaneous T cell lymphoma by inhibiting constitutively enhanced NF-κB activity of malignant T cells. Hence, DMF counteracts the crucial pathogenic factor in cutaneous T cell lymphoma such as death resistance. Importantly, the DMF effects observed in vitro could also be confirmed in vivo in a murine xenograft model [18]. The DMF-mediated induction of apoptosis in malignant T cells did not only inhibit tumor growth but also prevented cutaneous T cell lymphoma spreading into distant organs [18].

Recently, Zhao et al. [28] identified MCPyV small T antigen-induced non-canonical NF-κB signaling as an essential tumorigenic pathway in MCC. MCPyV small T antigenes uniquely activated non-canonical NF-κB, instead of canonical NF-κB signaling, to evade p53-mediated cellular senescence. Through its large T stabilization domain, MCPyV small T antigene-activated non-canonical NF-κB signaling by the induction of H3K4 trimethylation-mediated increased in NF-κB2 as well as in RelB transcription and also by promoting *NFKB2* stabilization and activation through F-box and WD repeat domain-containing 7 gene inhibition [28]. Non-canonical NF-κB signaling was required for senescence-associated secretory phenotype cytokine secretion promoting the proliferation of MCPyV small T antigenes. MCPyV-positive MCC cell lines and tumors showed non-canonical NF-κB pathway activation and senescence-associated secretory phenotype gene expression; the inhibition of non-canonical NF-κB signaling prevented MCC cell growth in vitro and in xenografts [28]. 

In virus-negative MCC cell lines, the tumor mutational burden is high (on average, 44.5 mut/Mbp), mutational patterns are strongly associated with UV-induced DNA damage, and many coding mutations of cancer-related genes do exist [32]. Inspired by the aforementioned data, we aimed to perform experiments by investigating for first time the effect of DMF on MCC cell lines and focusing on MCPyV-negative MCC cells. Consistent with the existing data to these substances we were able to observe a significant impact on the cell viability as well as the proliferation for all three cell lines. In the cytotoxicity assays of the cell lines, a strong effect was obtained from the treatment with DMF (10 µmol/L–200 µmol/L) for the two longer treatment durations. This finding is in agreement with the data of Nicolay et al. [18], which show a significant effect when cells from cutaneous T-cell lymphoma were treated with DMF for 48 h. When considering the treatment periods, it was visible that cell viability decreased with increasing treatment time in all three cell lines. There were also differences between the cell lines; when looking at the IC50_48 h_, the cell line MCC26 seemed to be more sensitive to DMF. This is in line with the fact that it has been previously shown that DMF leads to a time-dependent reduction in cell viability, which is cell-specific in extent [24]. A significant reduction in the cell viability of melanoma cells by DMF was also reported by Takeda et al. [19].

The three BrdUs performed with DMF all showed similar trends regarding the results. There were very significant reductions (*p* ≤ 0.0001) in proliferation for all cell lines and treatment periods, with all three showing very significant reductions (*p* ≤ 0.0001) below 10%. For the 24 h and 48 h treatment periods, strong reductions to under 5% were evident at relatively low concentrations starting at 100 µmol/L (MCC14.2, MCC26) or 150 µmol/L (MCC13). Similar to our data, where one could see a tendency toward time dependent PR reduction and a dosage-dependent PR reduction, some data suggest that DMF induces a concentration-dependent reduction in proliferation in melanoma cells as well as in human colorectal adenocarcinoma cell lines, similar to our results [15,20]. A temporal dependence was also observed, which is consistent with the trend of our results [15]. However, no cytotoxic effect was detected in either study using the LDH assay [15,20]. The results of Loewe et al. [22] also showed reduced proliferation after treatment with DMF, with 84 µmol/L determined as the concentration at which cell growth was fully inhibited. This concentration is comparable to the concentration at which we detected PRs below 5% in two of our cell lines.

Notably, this pilot study providing only preliminary results includes some limitations that have to be addressed in future investigations in this field. First of all, our investigations focused on MCPyV-negative MCC, however, future studies should also include virus-positive MCC cell lines. Moreover, additional control cell lines (e.g., keratinocytes) and functional experiments with respect to the NF-κB signaling should be included. Regarding the issue of drug resistance development, several rounds of DMF treatments could be performed on the eventual surviving MCC cells. If drug resistance is consistently and easily occurring, DMF might be combined with additional drugs. NF-κB signaling is known to be regulated by the p38 stress-signaling pathway. It would be worth seeing whether NF-κB as well as p38 signaling is downregulated by DMF. If yes, then p38 inhibitors could possibly potentiate or even act synergistically with the DMF treatment, resulting in a better outcome and a decrease in the emergence of drug resistant cancer cells. Moreover, in vitro cell invasion assays are recommendable for future studies to exclude that DMF induce a paradoxical increase in cell invasion in the MCC cell lines used.

## 5. Conclusions

Despite current immunotherapy regimens, there is still a need for other treatment approaches for MCC patients. Using different MCPyV-negative cell lines, we have investigated the in vitro effect of DMF, a substance for which an antineoplastic effect in combination with other tumors has been shown. A reduction in cell viability and an inhibition of proliferation were observed, with DMF proving effectiveness at doses starting from 10 µmol/L or 50 µmol/L depending on the assay and cell line. For the treatment duration of the cell lines with the substrate, a dependent effect was clearly shown in the MTT-assays, as well as a tendency in the BrdU-assays. Repeating the tests and extending them to include supplementary procedures could provide further insights. Even with the existing data, it is becoming apparent that FAE such as DMF should be investigated further as a promising therapeutic agent for MCPyV-positive MCC as well. 

## Data Availability

Derived data supporting the findings of this study are available from the corresponding author on reasonable request.
